# Feasibility and acceptability of using medical and nursing students to provide Implanon NXT at the community level in Kinshasa, Democratic Republic of Congo

**DOI:** 10.1186/s12905-020-00993-9

**Published:** 2020-06-24

**Authors:** Julie H. Hernandez, Pierre Akilimali, Annie Glover, Jane T. Bertrand

**Affiliations:** 1 Department of Health Policy and Management, Tulane University School of Public Health and Tropical Medicine, 1440 Canalt St, Suite 1900, New Orleans, LA 70112 USA; 2 Kinshasa School of Public Health, University of Kinshasa, Kinshasa, Democratic Republic of the Congo

**Keywords:** Implants, Task-shifting, Community-based distribution, Feasibility and acceptability, Democratic Republic of Congo

## Abstract

**Background:**

The use of implants has steadily increased in Kinshasa since 2013 but clinic-based access to this family planning method is limited due to distance and costs barriers. The objective of this study was to examine the feasibility and acceptability of providing Implanon NXT at the community level using medical and nursing students (M/N) as distributors, as part of a strategy to improve contraceptive uptake in the Democratic Republic of Congo.

**Methods:**

A cohort of 531 women who chose to receive Implanon NXT from a M/N student during community-based campaign days participated in three rounds of a quantitative survey administered at the time of insertion of the method, and at 6 and 12 months later. We conducted descriptive analysis to assess the feasibility and acceptability of providing the method through M/N students in terms of method choice, user profiles, contraceptive history, experience with insertion and side effects, continuation / discontinuation of the method, and overall satisfaction with FP services as well as students’ preparedness and capacity to safely offer the method, and their satisfaction with the experience..

**Results:**

The study demonstrated the feasibility of training students for community-based provision of Implanon NXT and 95% of them were satisfied with their experience. Acceptability of both the method and the service delivery strategy was high among participants, including among young and first-time contraceptive users. Out of the 441 women with a known outcome at 12 months, 92% still had Implanon NXT inserted, despite some of them reporting experiencing side effects. The vast majority (79%) would “strongly recommend” obtaining NXT from a M/N student if a friend wanted to avoid pregnancies.

**Conclusions:**

The provision of Implanon NXT at the community-level is a promising solution to address some of the barriers to accessing this method for women living in Kinshasa. However, strengthening pre-insertion counseling, particularly on expected side-effects and the possibility of early removal, is necessary to increase informed choice for the women and potentially limit method discontinuation.

## Background

In countries where contraceptive prevalence remains low and access to facility-based healthcare is difficult because of costs, transportation or health systems weaknesses, task-shifting the provision of family planning services to lower cadre of health workers (i.e. not doctors or nurses) operating at the community level has proven successful in a variety of settings [1, 2]. In Sub-Saharan Africa, a majority of these initiatives have focused on task-shifting the provision of injectable contraceptives and almost all of the pilot studies and following scale-up projects have used lay community health workers without previous medical training to carry out the task-shifting efforts [3–7].

Contraceptive prevalence in Kinshasa, Democratic Republic of Congo (DRC), is among the lowest on the continent (23.4% among married women as of 2017 [8]). Despite community-based provision of Family Planning (FP) services being a keystone of the National Strategic Plan for Family Planning (2014–2020) [9], the Ministry of Health does not permit the use of non-formally trained health workers for provision of contraceptives other than pills, condoms and Cyclebeads. In 2015 however, a pilot project leveraged one exception to that rule and successfully tested the feasibility and acceptability of using medical and nursing school (M/N) students to provide DMPA-SC at the community level [10].

These positive evaluations sparked new opportunities for task-shifting in DRC, with a particular emphasis on the potential of using M/N students in community-based provision of FP services [11]. Of particular interest was the feasibility and acceptability of using those same students to provide implants – which is one of the most commonly used methods in the country – at the community level. Contrary to other Sub-Saharan Africa countries, injectables are not a preferred method in DRC, with the method mix being largely dominated by implants and condoms, each comprising 29% of all FP users [8]. However, up until 2017, implants in DRC (mostly the 5-year two-rods Jadelle) were only available at healthcare facilities, and at a high cost to potential clients due to commodity costs, registration and other fees. In 2017, the introduction in DRC of Implanon NXT (NXT), a new model of single-rod implant developed by MSD and preloaded in a disposable applicator [12], in combination with the potential of using M/N students to provide contraceptives, created the opportunity to pilot the provision of the new method by this cadre.

There are comparatively few existing studies of task-shifting the provision of implants at the community level in Sub-Saharan Africa, possibly because of the widespread preference for injectables in these countries and the high level of required skills for implants insertion and removal. However, available evidence points towards the feasibility and accessibility of using trained health workers to provide implants at the community level [13]. Studies in Nigeria [14] and Ethiopia [15] were initiated to explore opportunities for expanding the method mix available to women in communities where the number of implant users was negligible. Unlike previous efforts, this paper presents evidence from a pilot study that was conducted to evaluate possible opportunities and barriers to expanding access to a highly demanded method in DRC, as part of strategies to address high unmet need for contraception in the study setting [8]. In particular, the paper examines the feasibility and acceptability of providing Implanon NXT at the community level using M/N students as providers as part of a pilot project to increase contraceptive uptake in the Democratic Republic of Congo.

## Methods

### Pilot provision of NXT at the community level

In November 2016, 48 students from nine M/N schools were recruited and trained in the community-level provision of a full range of free contraceptive methods, including condoms, pills, CycleBeads™, DMPA-SC and Implanon NXT. One campaign day was organized at or near the compounds of six health centers in three urban, semi-rural, and rural health zones of Kinshasa and publicized beforehand in the community. Women coming to the campaign received counseling on all FP methods from the M/N students, who either provided them with their method of choice for free, or referred those who chose methods that required expertise to administer (such as five-year implant or Jadelle, IUD, or sterilization) to health facilities. The partnering of M/N students operating at the community-level with referral facilities met two key requirements: (1) providing access to the full range of contraceptives (including IUDs, other implants such as Jadelle and Levoplant and permanent methods, only offered by clinical staff), and (2) strengthening quality of care by offering counseling and services for side-effect management and implant removal.

### Data collection

Routine service statistics were collected using national health system reporting forms to record the age, marital status and method (if any) chosen by the clients. Women who chose Implanon NXT were asked to participate in a survey administered by trained interviewers who recorded their responses using the OpenDataKit (ODK) app on Android smartphones. These initial acceptors were also asked if they would agree to be contacted a few months later for a follow-up interview. Those who agreed gave their contact information to the interviewers, who tracked and re-interviewed them at six (May 2017) and twelve (November 2017) months. All women were identified using a unique Identification Number to follow their responses between datasets.

Both the initial and follow-up surveys included questions about the women’s socio-economic characteristics, family planning history, contraceptive method preferences, experience with Implanon NXT insertion, and evaluation of the services provided by M/N students. In addition, the six- and twelve-month follow-up surveys asked about side effects experienced since NXT insertion, method continuation, and future FP intentions. Surveys were designed to give women the opportunity to evaluate separately NXT as a method and the use of M/N students as community-based distributors (See Supplementary files). Because pregnancy was considered a critical adverse outcome (indicative of either method failure or service delivery failure), the local team contacted the 8 women who reported becoming pregnant after receiving NXT from a student and completed in-depth interview with each of them to understand their circumstances. A separate survey targeted all 48 M/N students to collect their socio-demographic characteristics, as well as data on their training and their experience as Community-Based Distributors (CBD). (See Supplementary files).

### Data analysis

We assessed feasibility of using M/N students as Implanon NXT providers at the community level by looking at their satisfaction with the training, comfort with NXT insertion during the pilot, successful provision of NXT (number of implants inserted and reported adverse reactions at insertion site from the users), satisfaction with their experience and likelihood to recommend participating in similar activities to fellow students.

To examine acceptability of the approach among FP users, we considered indicators used by similar studies on acceptability of novel contraceptive technologies or delivery strategies [5–7]. These include women’s demographic characteristics, prior experience with modern contraception and concerns about community stigma as possible factors affecting contraceptive use. We also considered expected and experienced pain as well as other adverse reactions during and after insertion, intention to use and actual method continuation, as well as willingness to recommend the method and / or having a M/N student as FP service provider to their peers as key indicators of acceptability. We further examined the reported satisfaction of NXT acceptors with both the method and the service delivery strategy.

All datasets were analyzed in Stata 16.0. We obtained frequencies of acceptors’ socio-demographic profile, experiences with and evaluation of NXT, and the FP services provided by the student CBDs. Where relevant, we conducted statistical tests to determine the significance of associations between participants’ socio-demographic characteristics and selected indicators.

## Results

### Method choice and cohort description

Based on routine service statistics reported by the M/N students at the end of the pilot, out of 909 FP clients served over six campaign days, 76.7% (*N* = 697) chose Implanon NXT (followed by condoms^1^: 16.8%, DMPA-SC: 9.8%, CycleBeads™: 6.7% and the pill: 3.2%). There was no statistically significant difference in the distribution of acceptors of Implanon NXT and other methods by age.

**Fig. 1:**
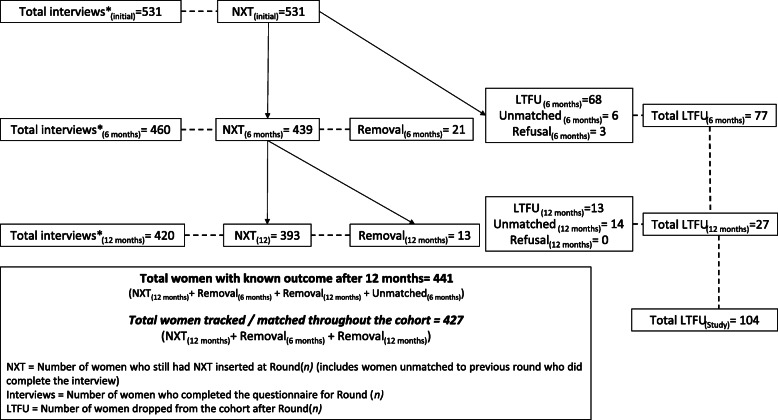
Cohort outcomes

A total of 531 women agreed to participate in the initial acceptor survey in November 2016. Six months later, 460 women completed a follow-up survey, including six who could not be matched to the initial acceptors database and were dropped from the cohort. Out of the 454 women remaining in the cohort, 420 were interviewed at 12 months (including 14 unmatched cases). Overall, out of the initial 531 acceptors, 441 women had a known outcome (and 427 could be traced from the insertion through the first year of NXT use) and 104 (19.5% of the initial cohort) were lost to follow-up, refused a follow-up interview, or could not be matched to their initial survey data. Figure 1 details the denominators for each round of surveys and the outcomes throughout the cohort.

### Feasibility of using M/N students to provide Implanon NXT at the community level

All M/N students felt they had been either very well (87.5%) or well (12.5%) prepared to provide Implanon NXT after completing training. Students were able to insert on average 14.5 Implanon NXT during the pilot study. Among the 531 women who received Implanon NXT and participated in the initial survey, only 5.7% (*N* = 30) reported a skin irritation at the insertion site and the majority of them (21/30) indicated that the reaction was minor and disappeared after a day or two.

In the post intervention survey, students reported that it took on average 3.1 insertions for them to be completely comfortable with NXT provision. Majority of them reported being satisfied (20.2%) or very satisfied (74.4%) with their experience, and all M/N students would either encourage (8.5%) or strongly encourage (91.5%) their peers to participate in future community-based distribution activities (Table 1).

**Table 1:** M/N student self-assessment of CBD^*^ experience

**Felt prepared after training to distribute contraceptives in community**	***N*** **= 48 (%)**
Very well prepared	42 (87.5)
Fairly well prepared	6 (12.5)
Not very well prepared	0 (0.0)
Not at all prepared	0 (0.0)
**Adequacy of practice before working alone in community**	***N*** **= 48 (%)**
Fully adequate	27 (56.3)
Somewhat adequate	20 (41.7)
Not really adequate	1 (2.1)
Not at all adequate	0 (0.0)
**How worried/anxious before inserting Implanon NXT for the first time**	***N*** **= 48 (%)**
Very anxious	6 (12.8)
Somewhat anxious	17 (36.2)
Not very anxious	8 (17.0)
Not anxious at all	16 (32.0)
Does not remember	1 (2.1)
**Concerns about inserting Implanon NXT**	***N*** **= 42 (%)**
Hurting the woman	11 (26.2)
Improper disinfecting / poor preparation of insertion	10 (23.8)
Taking too long to do the insertion	3 (7.1)
Not advising woman about side effects	0 (0.0)
Other	22 (52.4)
**Number of insertions until comfortable, mean (Standard Deviation)**	**3.1 (1.95)**
**Experienced stock-out of contraceptive supplies at least once**	**38 (80.9%)**
**Experience stock-out of Implanon NXT**	***n*** **= 38 (%)**
Often	14 (36.8)
Sometimes	9 (23.7)
Once	4 (10.5)
Never	11 (29.0)
**Satisfaction with experience as a CBD**	***N*** **= 48 (%)**
Very satisfied	35 (74.5)
Somewhat satisfied	8 (17.0)
Somewhat unsatisfied	5 (8.5)
Very unsatisfied	0 (0.0)
**Would encourage another medical or nursing student to become CBD**	***N*** **= 48 (%)**
Would strongly encourage	44 (91.5)
Would encourage	4 (8.5)
Would discourage	0 (0.0)
Would strongly discourage	0 (0.0)

*CBD* Community-Based Distributors

### Profile of Implanon NXT acceptors

The average age of the initial acceptors was 27 years, and 77.6% had at least attended secondary school. More than two-thirds (68.0%) were married or in union, and all but one woman had at least one child, with the mean number of children being 3.5. About two-thirds (63.8%) held a job, with the majority being self-employed (88.2%).

Before receiving NXT, 62.5% of the women had used an FP method before, with withdrawal (61.5%), condoms (52.1%), injectables (26.8%), and pills (22.3%) being the most commonly reported methods (multiple responses allowed). However, about half (47.8%) of the NXT acceptors were entirely novice to modern contraceptive methods, and only 3% had previously used implants (Table 2).

**Table 2:** Profile of Implanon NXT acceptors

	Full sample at baseline	Completed study	Loss to follow-up	Significance test^**b**^
	***N*** = 531 (%)	***N*** = 427^a^ (%)	***N*** = 104 (%)	
**Age, mean (Standard Deviation)**	27.1 (6.2)	27.4 (6.3)	26.3 (5.5)	*p* = 0.121
**Last year of education attended**				
None	22 (4.1)	21 (4.9)	1 (1.0)	*p* = 0.117
Primary or less	86 (16.2)	64 (15.0)	22 (21.2)	
Secondary	412 (77.6)	332 (77.8)	80 (76.1)	
University	11 (2.1)	10 (2.3)	1 (1.0)	
**Employed**				
Yes	339(63.8)	149 (34.9)	43 (41.3)	*p* = 0.219
No	192(36.2)	278 (65.1)	61 (58.7)	
**Type of employment (*n*** **= 339)**				
Self-employed	299 (88.2)	249 (89.6)	50 (82.0)	*p* = 0.126
Employed by someone else	28 (8.3)	19 (6.8)	9 (14.8)	
Employed by a family member	12 (3.5)	10 (3.6)	2 (3.3)	
**Marital status**				
Civil marriage	4 (0.8)	3 (0.7)	1 (1.0)	*p* = 0.57
Customary marriage	71 (13.4)	58 (13.6)	13 (12.5)	
Religious marriage	11 (2.1)	11 (2.6)	0 (0.0)	
Lives in union	275 (51.8)	219 (51.3)	56 (53.8)	
Not in union	170 (32.0)	136 (31.9)	34 (32.7)	
**Has living children**	530 (99.8)	427 (100.0)	103 (99.0)	*p* = 0.196
Number of children, mean (Standard Deviation)	3.5 (2.5)	3.6 (2.7)	3.3 (2.0)	*p* = 0.225
**Contraceptive History**				
**Ever done anything to delay or avoid pregnancy?**	332 (62.5%)	268 (62.8)	64 (61.5)	*p* = 0.817
**Contraceptive methods used in the past** (*n* = 332)				
Withdrawal	204 (61.5)	159 (59.3)	45 (70.3)	0.105
Male condom	173 (52.1)	137 (51.1)	36 (56.2)	0.460
Injectables	89 (26.8)	72 (26.9)	17 (26.6)	0.961
Pill	74 (22.3)	54 (20.1)	20 (31.2)	0.055
Emergency contraception	31 (9.3)	27 (10.1)	4 (6.20)	0.345
MAMA^c^	20 (6.0)	16 (6.0)	4 (6.2)	0.933
Cyclebeads	11 (3.3)	7 (2.6)	4 (6.2)	0.144
Implants	10 (3.0)	8 (3.0)	2 (3.1)	0.953
Female condom	7 (2.1)	4 (1.5)	3 (4.7)	0.110
IUD	2 (0.6)	1 (0.4)	1 (1.6)	0.269
Male sterilization	1 (0.3)	1 (0.4)	0 (0.0)	na
Other	46 (13.9)	42 (15.7)	4 (6.2)	0.050
Recent history of contraceptive use				*p* = 0.96
Used modern method in the past 12 months	187 (35.2)	151 (35.4)	36 (34.6)	
Used traditional method in the past 12 months	119 (22.4)	94 (22.0)	25 (24.0)	
None	225 (42.4)	182 (42.6)	43 (41.3)	

^a^ 427 women completed the study, including those who removed Implanon after 6 months and were not interviewed at 12 months. Some participants’ Identification Number (ID) could not be matched to baseline IDs at Round 2 or 3; unmatched participants were dropped from the study and counted as lost to follow-up

^**b**^T-tests and chi squared tests to determine if significant differences exist between participants who stayed in the study for the full 12-months (or until removal of Implanon NXT) and participants lost to follow-up. All measurements taken at baseline

^**c**^Méthode de l’allaitement maternel et de l’aménorrhée (lactational amenorrhea method)

The perception of community support towards FP was split, with half of the women (48.9%) reporting that “most” or “almost all” people in their community were in favor of using modern contraceptives whereas the other half indicated that “almost no one” in their community supported the use of such methods. The most commonly mentioned negative perceptions were fear of future sterility and perceived promiscuity among FP users *(Data not shown in Tables)*. However, 81.2% of NXT acceptors were “not concerned at all” by community opinions when coming for a method. Among married women, 68.4% reported that their husbands knew and approved of their FP visit that day. The majority of women (66.7%) made the decision to come and receive FP services alone, and in 21.1% of the cases it was a joint decision with the husband (Table 3).

**Table 3:** Partner and community attitudes towards family planning

	Initial acceptors ***N*** = 531 (%)
**When you went looking for family planning services today, were you concerned that someone would see you and guess the reason for your presence?**	**N = 531**
Not concerned at all	431 (81.2)
Somewhat concerned	73 (13.8)
Very concerned	27 (5.1)
No response	0 (0.0)
**Does your husband/partner know that you came to the family planning consultation today?**	***N*** **= 361 (%)**
Yes	247 (69.4)
No	103 (28.5)
No response	11 (3.1)
**Does your husband/partner agree with you using a contraceptive method?**	***N*** **= 247 (%)**
Completely agrees	239 (96.8)
Somewhat agrees	7 (2.8)
Somewhat disagrees	1 (0.4)
Completely disagrees	0 (0.0)
Does not know / No answer	0 (0.0)
**Do you think your partner / husband would agree or disagree that you are using a contraceptive method?**	***N*** **= 103 (%)**
Completely agrees	52 (50.4)
Somewhat agrees	13 (12.6)
Somewhat disagrees	11 (10.6)
Completely disagrees	23 (22.3)
Does not know / No answer	4 (3.8)
**Who took the decision to come to this Family Planning consultation?**	***N*** **= 531**
Me alone	354 (66.7)
My husband / partner and myself (joint decision)	112 (21.1)
Friends / family / neighbors	104 (19.6)
Husband / partner	81 (15.3)
Healthcare staff	6 (1.1)
Community-based distributors	6 (1.1)
Other	4 (0.75)

### Satisfaction with the method and continuation

Regarding experience with receiving NXT, 24.3% of initial acceptors were “somewhat” and another 5.8% were “very” concerned prior to the insertion, with fear of pain during the procedure (77.9%) and fear of side effects (43.8%) being the most commonly mentioned sources of concern. A third of the women found the actual insertion “somewhat” (34.3%) or “very” (1.3%) painful. Immediately after Implanon NXT insertion, most women would either “recommend” (31.5%) or “strongly recommend” (67.2%) Implanon NXT to a friend who wants to avoid pregnancies (Table 3).

During the six-month follow-up survey, two-thirds of the women (63.0%) declared experiencing some side effects after Implanon NXT insertion, and that proportion remained similar at 12 months (57.6%). In both follow-up surveys, women reported amenorrhea [44.1% at 6 months and 48.8% at 12 months], irregular periods [35.9% at 6 months and 25.6% at 12 months], heavier bleeding than usual [21.4% at 6 months and 17.8% at 12 months] and abdominal pain [14.8% at 6 months and 13.7% at 12 months] as the most common side effects (Table 4 – All data based on women’s self-assessment).

Out of the 441 women with a known outcome at 12 months, 34 (7.7%) had opted to remove Implanon NXT (21 in the first 6 months and another 13 between 6 and 12 months after insertion) (Fig. 1). For the 427 women whose outcomes could be traced since insertion, there was no statistically significant difference in the distribution of those who discontinued and those who continued using Implanon NXT at 12 months by age, level of education, employment status, number of children, or contraceptive history (Table 2).

Women who discontinued using the method mostly reported experiencing side effects (particularly heavy or irregular bleeding) as the main reason for discontinuation (81.0% at 6 months and 92.3% at 12 months). A third of those who removed the method (28.6% at 6 months and 38.5% at 12 months) mentioned opposition from their partners; three women reported that they wanted to switch to another method and one indicated that she wanted to become pregnant. Apart from those who discontinued use, 27 women (6.6%) indicated that they thought about having NXT removed during the first year of use. This was also predominantly due to side effects (21 out of 27 respondents), and other people’s (but not specifically their partner’s) opposition (4 out of 27). However, the majority decided to tolerate the side effects (18 out of 27) or managed to convince the opposing person in order to continue using NXT. In two cases, women who wanted to discontinue kept the implant inserted because they did not know where to go or could not find someone competent for removal (Table 4).

There were eight cases of pregnancies (1.8% of 441 known outcomes) recorded during the pilot study. The follow-up interviews with those eight women indicated that they were in fact pregnant before NXT was inserted but that their pregnancies had not been detected by the students. Because pregnancy tests are not easily available in DRC, the students used a checklist of questions to rule out existing pregnancy but did not systematically administer a clinical test. The interviews suggested that, in three cases, the students did not properly complete the screening procedures, and in five cases women deliberately deceived the student, in the hope that the NXT insertion would trigger a spontaneous abortion). *(Qualitative data not shown in tables)*.

Overall, continuation of the method ranged between 76.6% (if all 90 women with no known outcome were assumed to have removed NXT) and 93.5% (if all 90 women were still using the method).

At the end of the pilot, 93.9% of the 407 women who still had Implanon NXT inserted declared that they would continue using the method in the future, mainly because of its effectiveness (83.2%), ease of use (35.1%), and comparatively minor side effects (17.8%). When asked how long they ideally would like to delay their next birth, those who were still using the method mostly reported between 1 to 3 years (54.8%) and 4 to 6 years (27.9%) with 15.7% wanting to be protected for more than 7 years. Only 1.7% of NXT users wanted to delay their next birth by less than a year (Table 4).

Satisfaction with the method remained constant throughout the pilot, and after a year, a similar proportion of women would “strongly recommend” (79.0%) or “recommend” (19.0%) NXT to a friend who wanted to protect herself from pregnancies. It is not possible to determine from available data if the higher percentage who would strongly recommend (67.2% at insertion vs. 79.0% at 12 months) is the result of bias among the women still included in the cohort at 12 months or an indication of the increased satisfaction of users over time (Table 4).

**Table 4:** Experience with Implanon NXT (insertion and first 12 months)

	Initial acceptors	6 months	12 months
	***N*** = 531 (%)	***N*** = 460 (%)	***N*** = 420^**a**^ (%)
**How concerned were you before receiving Implanon NXT**?			
Very concerned	31 (5.8)		
Somewhat concerned	129 (24.3)		
Not concerned at all	371 (69.9)		
**Specific concerns (*n*** **= 160) for those who reported being “somewhat” or “very” concerned**			
Fear of pain during or after insertion	115 (77.9)		
Fear of side effects	70 (43.8)		
Efficacy in preventing pregnancy	19 (11.9)		
Fear of future infertility	16 (10.0)		
Fear of birth defects	6 (3.8)		
Husband / partner does not agree	3 (1.9)		
More familiar with other methods	2 (1.3)		
Other	9 (5.6)		
**How painful was the insertion?**			
Not painful	342 (64.4)		
Somewhat painful	182 (34.3)		
Very painful	7 (1.3)		
Did you experience a skin reaction or irritation at insertion site?	30 (5.7)		
**How severe was this skin reaction? (n = 30)**			
Minor	21 (70.0)		
Moderate	8 (26.7)		
Serious	1 (3.3)		
		**6 months*N*** **= 460 (%)**	**12 months*N*** **= 420**^**a**^**(%)**
**Had side effects from Implanon NXT**		290 (63.04)	242 (57.62)
**Side effects**		***N*** **= 290 (%)**	***N*** **= 242 (%)**
Amenorrhea		128 (44.1)	118 (48.8)
Irregular periods		104 (35.9)	62 (25.6)
Frequent / heavy bleeding		62 (21.4)	43 (17.8)
Abdominal pain		43 (14.8)	33 (13.60)
Backaches		23 (7.9)	19 (7.90)
Weight loss		26 (9.0)	15 (6.20)
Headaches		8 (2.8)	11 (4.50)
Weight gain		17 (5.9)	9 (3.70)
Nausea / vomiting		16 (5.5)	7 (2.9)
Weakness / fatigue		9 (3.1)	6 (2.5)
Pain at insertion site		1 (0.3)	4 (1.7)
Acne		1 (0.3)	2 (0.8)
Breast tenderness		4 (1.4)	1 (0.4)
Decreased libido		2 (0.7)	1 (0.4)
Vaginal dryness		1 (0.3)	0 (0.0)
Other		19 (6.6)	17 (7.0)
**Since receiving Implanon NXT, have you become pregnant?**		7 (1.50)	1 (0.2)
**How long would you like to wait before having your next child?**			*N* = 420 (%)
Less than 1 year		Non Applicable (NA)	7 (1.7)
1–3 years		NA	230 (54.8)
4–6 years		NA	117 (27.9)
7–9 years		NA	24 (5.7)
10 or more years		NA	42 (10.0)
**For women who continued using Implanon NXT**			
**Reasons for continuing with Implanon NXT**		***N*** **= 407 (%)**	***N*** **= 382 (%)**
Long term protection		312 (76.7)	NA
Effective		165 (40.5)	318 (83.2)
More effective than previous methods		NA	52(13.6)
Easy to use		143 (35.1)	134 (35.1)
Few side effects		77 (18.9)	68 (17.8)
Painless		52 (12.8)	NA
Easy to hide		28 (6.9)	50 (13.1)
No need to go to health facility		7 (1.7)	8 (2.1)
No need to remember dates		17 (4.2)	49 (12.8)
Other		13 (3.2)	10 (2.6)
**At any point, did you want to have Implanon NXT removed?**		NA	21 (5.2)
**Reasons for wanting to get it removed**			***N*** **= 27**
Side effects		NA	21 (77.8)
Partner opposition		NA	1 (3.7)
Opposition from another person		NA	3 (11.1)
Want to get pregnant		NA	1 (3.7)
Other		NA	3 (11.1)
**Why did you keep Implanon NXT if wanted it removed?**			***N*** **= 27 (%)**
Decided to tolerate side effects		NA	18 (66.7)
Convinced the opposing person		NA	3 (11.1)
Does not know where to find preferred method		NA	1 (3.7)
Does not know where to get Implanon NXT removed		NA	1 (3.7)
Tried to get it removed but no one was competent to do so		NA	1 (3.7)
Other		NA	5 (18.5)
**Intention to continue using Implanon NXT in the future**			***N*** **= 407 (%)**
Yes		NA	382 (93.9)
No		NA	21 (5.2)
Does not know		NA	4 (1.0)
**For women who had Implanon NXT removed**			
**Reasons removed Implanon NXT**		***N*** **= 21 (%)**	***N*** **= 13 (%)**
Side effects		17 (81.0)	12 (92.3)
Partner disapproved		6 (28.6)	5 (38.5)
Other		5 (23.8)	1 (7.7)
Wanted to get pregnant		1 (4.8)	0 (0.0)
Prefer another method		3 (14.3)	0 (0.0)
**Overall satisfaction with Implanon NXT?**			
	**Initial**	**6 months**	**12 months**
**Would recommend Implanon NXT to a friend**	***N*** **= 531 (%)**	***N*** **= 460 (%)**	***N*** **= 420 (%)**
Strongly recommend	357 (67.2)	404 (87.8)	332 (79.0)
Recommend	167 (31.5)	39 (8.5)	80 (19.0)
Does not recommend	5 (1.0)	5 (1.1)	5 (1.2)
Indifferent / does not know	2 (0.4)	3 (0.7)	3 (0.7)

^**a**^ 427 women completed the study, including those who removed Implanon after 6 months and were not interviewed at 12 months. Some participant Identification Number (ID) could not be matched to baseline IDs at Round 2 or 3; unmatched participants were dropped from the study and counted as lost to follow-up

### Satisfaction with M/N students as contraceptive providers

Regarding the services provided by the M/N students, 96.4% of the women indicated that the providers were “very comfortable” while doing the insertion and all women found them “respectful” or “very respectful” during the visit. While 73.3% of all acceptors did not realize at first that the person inserting NXT was a student, out of the 142 women who identified them as such, the vast majority (94.4%) were (very) comfortable with receiving the services from the students rather than from a fully trained doctor or nurse.

In general, the acceptors felt that they received adequate counseling from the M/N students, with 74.6% reporting that they received “very clear explanations” (and 11.1% “adequate explanations”) regarding possible side-effects of the method. In addition, 88.1% reported that they would go to the health center recommended by the CBD if necessary (12.8% indicated they would come back to see the CBD, and 7.9% stated they would visit a different healthcare provider). However, when specifically asked how long they were informed to keep the implant inserted, 76.5% of the acceptors remembered being told they had to keep it for 3 years, with only 16.9% knowing they could have it removed earlier (and 6.6% not remembering) (Table 5).

**Table 5:** Experience receiving the method from a M/N^a^ student

**Perceived comfort of CBD**^b^**with inserting Implanon NXT**	***N*** **= 531 (%)**
Very comfortable	512 (96.4)
Somewhat comfortable	11 (2.1)
Somewhat uncomfortable	7 (1.3)
Very uncomfortable	1 (0.2)
**CBD provided clear explanations of Implanon NXT side effects**	
Very clear explanations	396 (74.6)
Adequate explanations	59 (11.1)
Unclear explanations	29 (5.5)
No explanation	47 (8.9)
**CBD told acceptor where to go with question or if experience side effects**	438 (82.5)
**Where would you go with questions or if experience side effects?**	
Health center recommended by the CBD	468 (88.1)
Recontact same CBD	68 (12.8)
Another healthcare provider	42 (7.9)
Families, friends, neighbors	0 (0.0)
Husband / partner	0 (0.00)
Other	49 (9.2)
Do not know	14 (2.6)
**How long did CBD tell you to keep Implanon NXT? (12-month survey)**	***N*** **= 378 (%)**
Must keep it in for 3 years	289 (76.5)
Can have it removed before	64 (16.9)
Does not know / remember	25 (6.6)
**Level of satisfaction with information and counsel from student CBD**	
Very satisfied	490 (92.3)
Somewhat satisfied	37 (7.0)
Somewhat unsatisfied	3 (0.6)
Very unsatisfied	1 (0.2)
**Level of satisfaction with insertion of Implanon NXT by student CBD**	
Very satisfied	508 (95.7)
Somewhat satisfied	21 (4.0)
Somewhat unsatisfied	2 (0.4)
Very unsatisfied	0 (0.0)
**Global satisfaction with service provided by student CBD**	
Very satisfied	512 (96.4)
Somewhat satisfied	15 (2.8)
Somewhat unsatisfied	4 (0.8)
Very unsatisfied	0 (0.0)
**Knew CBD was a M/N student before receiving Implanon NXT**	142 (26.7)
**Comfort with a student inserting Implanon NXT rather than doctor or nurse**	***N*** **= 142 (%)**
Very comfortable	129 (90.9)
Somewhat comfortable	5 (3.5)
Somewhat uncomfortable	8 (5.6)
Very uncomfortable	0 (0.0)

^**a**^M/N student: Medical / Nursing student

^**b**^*CBD* Community-Based Distributor

## Discussion

The high levels of satisfaction reported by both users and providers of Implanon NXT are indications of the acceptability and potential expansion of both the new method and community-based distribution through M/N students as strategies to increase contraceptive uptake in DRC. In addition, user profiles, experience with insertion and method continuation suggest high acceptability. The large number of women choosing Implanon NXT, in settings where they could receive any method immediately and for free, is consistent with both existing data regarding method preferences in DRC, where use of implants among married women increased from 4% in 2014 to 19% in 2019 (PMA2020 Data Lab), and results from similar studies in Sub-Saharan Africa [13–15] Some attributes of the NXT acceptors, such as being on average older, more educated, and more often married or living in union than the average FP user in Kinshasa [8] may have made them more amenable to the method for spacing their next birth. However, the fact that almost half of the NXT acceptors in this cohort had never used any modern contraceptive before suggests a high acceptability even among entirely novice users. The majority of acceptors also reported favorably on both their experience with the insertion and the first few months of using the method, despite frequently mentioning side-effects, particularly those related to changes in their menstrual cycle. This is encouraging considering that some studies suggest that these types of side-effects could be a determinant of method discontinuation [16, 17]. As noted by Polis and colleagues, it is important to distinguish between bleeding patterns women “prefer (including the potential for no bleeding change)” and what they are “willing to tolerate in exchange for the benefits of available contraceptive options” [18]. Findings from this cohort point towards the latter attitude among NXT acceptors.

The high level of continuation of Implanon NXT at 12 months (estimated between 76.6 and 93.4%) in this cohort compared to that of other studies conducted in Sub-Saharan Africa (under 40.0% in South Africa [19] could also indicate a high acceptability of the method in the long run. The high level of continuation is similar to that of the industrialized nations, including the United States [20], Europe [21] and Australia [22]) and calls for further investigation. It could be that the acceptors of the method kept using it because of its effectiveness and convenience, which outweighed experienced side effects. It could also be that the women had limited access to removal services (in terms of knowledge of availability and cost of removal) if they wanted to discontinue the method.

The findings of this paper also showed that using M/N students as community-based providers was entirely acceptable to FP users in Kinshasa, who generally gave positive evaluations of the services they received. This is consistent with findings from piloting of community-based provision of DMPA-SC [10, 23] in Kinshasa, and similar studies indicating that community-health workers could successfully be used to increase access to contraceptives throughout Sub-Saharan Africa [1, 2].

Results on the feasibility of this approach were also encouraging. Studies conducted elsewhere have demonstrated that lay health workers can provide adequate contraceptive services, including for injectables and implants, with minimum additional training [3, 4, 13–15], and it is thus not surprising that M/N students could successfully and safely provide the services. Following findings from the pilot study, the project team retrained the students to improve screening for pregnancy and anesthetic practices to reduce instances of providing the method to women when they are already pregnant and to minimize pain during insertion.

These findings imply that using M/N students presents a promising opportunity for the provision of an array of contraceptive methods, including Implanon NXT, at the community level [11, 24]. They reinforce results from previous studies conducted in DRC, where the model of using nursing school students as CBD is currently being replicated and institutionalized throughout the country [10]. The strategy is likely to contribute to ongoing efforts to improve contraceptive uptake in the country not only by adding a new method into method mix but also removing some access barriers associated with obtaining services from health facilities [25].

Findings from this pilot study also suggest that key programmatic issues related to quality of FP counseling must be addressed to ensure the safety and satisfaction of the NXT acceptors. Specifically, and consistent with findings from other environments [26], providers should be better trained to counsel women on side effects before insertion. This is particularly important in DRC where, due to limited reproductive health knowledge, erratic periods are often interpreted as a risk for infertility. In addition, providers should give clear information on the possibility of removing implant before the end of 3 years given that the duration may influence women’s choice of the method based on their fertility desires as well as decisions to continue or discontinue the method based on experiences using it.

Additional areas of improvements highlighted here are inherent to DRC’s fragile health system. The country has been plagued with contraceptive stockouts as demand steadily increased over the past few years, and addressing gaps in the supply chain will be crucial for the successful scaling up of the service provision model tested in this pilot study [24].

This study also has certain limitations. First, the study involved a convenience sample of women, which may limit the generalizability of the findings to all women in Kinshasa or DRC. The study was embedded in programmatic efforts to demonstrate the feasibility and acceptability of both the method and the service delivery model and did not include comparison groups to measure these concepts against other service delivery models.

Second, the intervention design offered NXT through single events organized within or near a health facility; it was not a full community-based distribution system where CBDs circulate in their neighborhoods to distribute contraception in an effort to address barriers to access for facility-based FP services (e.g. cost of services and transportation, wait time, concerns about providers attitude). Both the location (close to facility-based services) and the fact that the CBDs had at least started their nursing training might also have increased the acceptability of this service delivery strategy to the clients, since the pilot settings were reasonably close to “routine” facility-based services.

The provision of Implanon NXT and other methods for free further makes it impossible to compare the acceptability of the method and women’s choices observed in this pilot to other models where women must pay for contraception. Nonetheless, the findings show that most women still chose Implanon NXT despite the availability of other methods (apart from IUD and sterilization) at no cost. This further underscores the potential for implants to contribute to overall FP uptake in DRC. It further highlights the need for improved counseling of women to reduce levels of discontinuation of the method as a result of side effects.

## Conclusion

The provision of Implanon NXT at the community-level is a promising strategy for overcoming some of the barriers to accessing this preferred method for women living in Kinshasa. However, programs must strengthen pre-insertion counseling, particularly on expected side effects and early removal, to ensure women’s informed choice and to potentially reduce method discontinuation. There should also be a focus on improving availability of removal services, including putting in place the relevant infrastructure and training of providers, since the growing uptake of Implanon NXT will translate into increased need for safe removal or replacement services.

## Supplementary information

**Additional file 1.**

**Additional file 2.**

**Additional file 3.**

## Data Availability

The datasets generated and analyzed during the current study are not publicly available due to their continued use in ongoing research, including 24 months follow-up with women, and funding agencies requirements, but are available from the corresponding author on reasonable request.

## Abbreviations

CBD: Community Based Distributors
DMPA-SC: Depot-medroxyprogesterone acetate Sub-Cutaneous
DRC: Democratic Republic of Congo
FP: Family Planning
IUD: Intra-Uterine Device
LTFU: Lost-To-Follow Up
MAMA: Méthode de l’allaitement maternel et de l’aménorrhée (lactational amenorrhea method)
M/N: Medical / Nursing Schools
MSD: Merck Sharp & Dohme
NXT: Implanon NXT
ODK: Open Data Kit
PMA: Performance in Monitoring and Accountability

## Footnotes

1 *Routine service statistics provided the number of women who received condoms but did not distinguish between those who obtained it as their main method and those who received it for HIV/STI protection in addition to another method.*
