# Supplementary material for: Feasibility and acceptability of using medical and nursing students to provide Implanon NXT at the community level in Kinshasa, Democratic Republic of Congo
Source: BMC Womens Health. 2020 Jun 24;20:133. doi: 10.1186/s12905-020-00993-9 (PMC7315479; doi:10.1186/s12905-020-00993-9)
Supplement: Supplementary file 1 — Additional file 1. [file 12905_2020_993_MOESM1_ESM.docx]

**Appendix A. d.: Questionnaire: Medical /Nursing Student (M/NS) Providers of Implanon NXT**

| Interviewer code: | \|___\|___\|___\| | Date: DD/MM/YY | \|___\|___\|___\| | Outreach: Y/N |
| --- | --- | --- | --- | --- |

**SECTION 1: CHARACTERISTICS OF M/NS PROVIDERS**

| **NO.** | **QUESTIONS ET FILTRES** | **CATEGORIES CODEES** | **SKIP** |
| --- | --- | --- | --- |
|  | How old were you on your last birthday? | AGE IN COMPLETED YEARS __________ |  |
|  | Are you a student in medical or nursing school? | 1. Nursing school 2. Medical school |  |
|  | What is the name of your school/university? | 1. UNIKIN/FAC. MED. 2. BEL CAMPUS/FAC.MED. 3. ISTM KIN 4. ISSSK et FAC .MED Kimbanguiste 5. ITM. KINTAMBO 6. INPESS 7. ITM NDJILI 8. IEM KINKOLE 9. IEM MALUKU 10. ITM BON ACCUEIL Kimbaseke 11. Other (Specify on the next screen) |  |
|  | What is your current level in this school/university? | 1. Has not yet finished first year  2. Has completed first year  3. Has completed second year  4. Has completed more than second year  5. Other (Specify) |  |
|  | Gender of respondent (observe): | 1. Male  2. Female |  |
|  | What is your current zone of residence? | 1. Bandalungwa 2. Barumbu 3. Bumbu 4. Kalamu 5. Kasa-vubu 6. Kimbanseke 7. Kinshasa 8. Kintambo 9. Kisenso 10. Lemba 11. Limete 12. Lingwala 13. Maluku 14. Masina 15. Matete 16. Mont-Ngafula 17. Ndjili 18. Ngaba 19. Ngaliema 20. Ngiri-Ngiri 21. Nsele 22. Selembao |  |
|  | Are you currently married, or do you live with someone as if you were married? | 1. YES, CIVIL OR RELIGIOUS MARRIAGE 2. YES, CUSTOMARY/TRADITIONAL MARRIAGE 3. YES, LIVING IN UNION 4. NO, NOT LIVING IN UNION (SINGLE) | 🡪 Q109  🡪 Q110 |
|  | Do you have children? | 1. Yes  2. No |  |
|  | What is your religion?  **DO NOT READ RESPONSES** | 1. CATHOLIC 2. PROTESTANT 3. MUSLIM 4. PENTECOSTAL 5. KimbanguisT 6. EVANGELICAL CHURCH 7. OTHER (specify): ___________________________ |  |
|  | Do you have any type of job for which you earn money? | 1. Yes  2. No |  |
| 112. | Do you own a mobile phone for your personal use? | 1. Yes  2. No |  |
| 112a | How comfortable are you with sending and receiving text messages? | 1. Very comfortable (I send and received texts every day) 2. Somewhat comfortable (I send and receive texts every week) 3. Somewhat uncomfortable (I rarely send and receive texts) 4. Not comfortable at all   99. No response |  |
| **The following questions concern your activities as a Community Based Distributor since you have been recruited by ASD** | | |  |
| 113. | In what month and what year were you asked to participate in this project? | Month \|___\|___\| Year \|___\|___\|  99 Does not remember |  |
| 113a | Why did you decide to participate in this project?  (SEVERAL ANSWERS POSSIBLE. DO NOT READ THE ANSWERS BUT CHECK ALL MENTIONNED) | 1. I wanted to help my community 2. I wanted to know more about family planning 3. I wanted to acquire new technical skills for my work 4. I wanted to acquire human / social skills for my work (counseling, clients follow-up) 5. I wanted university credits 6. Other (Specify on the next screen)   99. No answer |  |
| 114 | Before participating in this activity, did you receive any training on family planning at your school or your university? | 1. Yes 2. No   999 Doesn’t know / Doesn’t remember |  |
| 115 | Did you attend the training facilitated by ASD on the distribution of contraceptive methods at the community level? | 1. Yes 2. No   999 Don’t know / Don’t remember | 🡪Q117  🡪Q118 |
| 116 | Why didn’t you attend this training? | 1. Didn’t know about the training 2. Had other commitments (university, family, etc.) 3. Thought the training was optional 4. Attended a similar training from a different institution   999 DON’T KNOW/ NO ANSWER |  |
| 117 | How many days did the training last? | ___ NUMBER OF DAYS (OF TRAINING)  999 Don’t remember |  |
| 118 | How well would you say the training prepared you to be effective as a community based distributor of contraceptives? Would you say you were well prepared, somewhat prepared, somewhat unprepared, or not prepared at all?  (READ RESPONSES) | 1. Well prepared for this work 2. Somewhat prepared for this work 3. Somewhat unprepared for this work 4. Not prepared at all for this work   999 No answer |  |
| 119 | On what topics would you have liked to receive more training or more information?  (SEVERAL ANSWERS POSSIBLE. DO NOT READ THE ANSWERS BUT CHECK ALL MENTIONNED) | 1. Contraceptive methods and how they work 2. How to counsel women on secondary effects 3. How to insert an implant (NXT) 4. How to take out an implant (NXT) 5. How to recruit new acceptors at the community level 6. How to present the methods during community meetings 7. How to present the project to Health Zone staff 8. How to create and maintain relations with the healthcare centers 9. How to collect service statistics on the quantity of methods distributed 10. Other (Specify): _____   999. No response | 🡪Q120  🡪Q121  🡪Q119  🡪Q119  🡪Q119  🡪Q119  🡪Q119  🡪Q119  🡪Q119  🡪Q119  🡪Q119 |
| 120 | On which methods would you have liked to receive more training or more information?  (SEVERAL ANSWERS POSSIBLE) | 1. Cyclebeads 2. Male condoms 3. Female condoms 4. COC Pill 5. POP Pill 6. Sayana Press 7. Implanon NXT 8. Other (Specify on the next screen)   999 No response |  |
| 121 | Have you been able to practice inserting Implanon NXT enough during the training (before doing it at the community level) or was the practice not enough?  *Would you say the practice you got during training was …* | 1. The practice during training was completely adequate 2. The practice during training was somewhat adequate 3. The practice during training was somewhat inadequate 4. The practice during training was completely inadequate   999 No answer |  |
| 122 | Before starting the community-based distribution / during the practice session (stage), did you practice insertion on a mannequin or on a living person ? | 1. On a mannequin  2. On a living person  3. On a mannequin and a living person  4. On neither of these  999. No response |  |
| 122 | Before starting the community-based distribution / during the practice session (stage), how many Implanon NXT insertion did you perform? | Number of insertions performed: \|___\|___\|  999 Does not remember |  |
| 123 | After you received the training, did you participate in the distribution activities contraceptive and insertions of Implanon NXT? | 1. Yes  2. No | 🡪 Q124  🡪 Q125 |
| 124 | Why didn’t you participate in the distribution activities?  (DO NOT READ RESPONSES. SELECT ALL THAT APPLY) | 1. Had other university commitments 2. Had other personal / family obligations 3. Was not informed of the activities’ schedule 4. Technical problem (missing products or supplies) 5. Did not feel prepared enough to meet the community 6. Did not feel prepared enough to present Implanon NXT 7. Did not feel ready to perform an insertion 8. Fear of reactions from the community 9. Other: __________________________   999. Don’t know / No answer | 🡪 Fin du questionnaire |
| 125 | Where did the distribution activities in which you participated take place?  (READ ALL THE ANSWERS AND SELECT ALL THAT APPLY) | 1. Within a health center / hospital  2. In a community place (market, church, community center)  3. At your school / your university  4. At clients’ homes  5. Other (Specify) :_______  999 No answer |  |
| 126 | What activities did you perform at least once during the distribution campaign?  (SELECT ALL THAT APPLY) | 1. Informal group talk 2. Individual counseling 3. Implanon NXT insertion 4. Sayana Press injection 5. Referral to a health center / hospital 6. Other (Specify on the next screen)   999 No response | 🡪Q126 |
| 127 | During the community-based distribution activities (after the training), approximatively how many Implanon NXT did you insert?  IF THE CBD DOES NOT REMEMBER / IS NOT SURE? ASK THEM TO GIVE AN ESTIMATE | Number of insertions performed: \|___\|___\|  999 Does not remember |  |
| 128 | To confirm, you received training on Implanon NXT NXT, but you never performed any insertion to women in the community over the past 6 month? | 1. Yes, I received the training but never performed NXT insertion  2. No, I received the formation but I did perform NXT insertion | 🡪 Q128  🡪 Revoir Q125(6) |
| 129 | Why did you not perform any NXT insertion to women in the community?  (SEVERAL RESPONSES POSSIBLE. DO NOT READ THEM BUT CHECK ALL THAT APPLY) | 1. No woman chose Implanon NXT as a method  2. I was not comfortable enough to perform an insertion  3. The women were not comfortable with me performing the insertion  4. I never received / I was stocked out for Implanon NXT  5. Other (Specify: ___________________)  999. No response |  |

**SECTION 2: M/NS PROVIDERS’ EXPERIENCE WITH THE PROGRAM**

| **No.** | **QUESTIONS AND FILTERS** | **CODING CATEGORIES** | | | | | | | | | | **SKIP** |
| --- | --- | --- | --- | --- | --- | --- | --- | --- | --- | --- | --- | --- |
| ***These questions will only be asked to M/NS students who performed in at least one day of Implanon insertion activities***  **Next I’d like to ask you a few questions about your involvement in this program in which medical and nursing students distribute contraceptive methods including Implanon NXT at the community level.** | | | | | | | | | | | | |
| 201 | I will read you a list of activity, and for each of them I would like you to tell me if you were very comfortable, somewhat comfortable, not very comfortable or very uncomfortable in performing them in the community |  | **Very comfortable** | | **Comfortable** | | **Not very comfortable** | | **Very uncomfortable** | | **No response** |  |
|  |  | Recruiting acceptors |  | |  | |  | |  | | 999 |  |
|  |  | Group talks / Counseling |  | |  | |  | |  | | 999 |  |
|  |  | Explaining side effects |  | |  | |  | |  | | 999 |  |
|  |  | Inserting Implanon NXT |  | |  | |  | |  | | 999 |  |
|  | Were you supervised in person during your contraceptive distribution activities? (for instance, somewhat from ASD, PNSR, or Tulane) | 1. Yes  2. No  999 Don’t know / Not sure | | | | | | | | | | 🡪 Q203  🡪 Q204 |
|  | Did you find this supervision very useful, somewhat useful, not very useful, or not useful at all to do your travail? | 1. Very useful 2. Somewhat useful 3. Not very useful 4. Not useful at all   999 Don’t know / No opinion | | | | | | | | | |  |
|  | During your distribution activities, did you contact a supervisor/health professional to help you solve any question? | 1. Yes  2. No  999 Don’t know / Not sure | | | | | | | | | | 🡪 Q205  🡪 Q206  🡪Q206 |
|  | Who did you contact?  SELECT ALL THAT APPLY | 1. A teacher / Focal point from school/university 2. ASD staff 3. PNSR staff 4. Tulane staff 5. Health center staff 6. Someone else (Specify) : | | | | | | | | | |  |
|  | Overall, did you find the supervision of your activities adequate? | 1. Yes, completely adequate (no need for more) 2. Yes, quite adequate (but more would have been needed for certain aspects) 3. No, not very adequate (really need a supervisor) 4. There is too much supervision 5. 999. Don’t know / No opinion | | | | | | | | | |  |
|  | After you started working as a community based distributor, did you lack any product to distribute (i.e., did you experience stock outs?) | 1. Yes 2. No   999. Don’t remember/ Not sure | | | | | | | | | | 🡪Q208  🡪Q212 |
|  | Please, detail for each method if you have experienced stock outs once, a few times, frequently or never. | **208a. Pill** | | **208b. Condoms** | | **208c. Cycle beads** | | **208d. Sayana Press** | | **208e. Implanon NXT** | |  |
|  |  | Once | | Once | | Once | | Once | | Once | |  |
|  |  | 2 to 5 times | | 2 to 5 times | | 2 to 5 times | | 2 to 5 times | | 2 to 5 times | |  |
|  |  | More than 5 times | | More than 5 times | | More than 5 times | | More than 5 times | | More than 5 times | |  |
|  |  | Never | | Never | | Never | | Never | | Never | |  |
|  | The last time you experienced a stock out,, did you report the stock out to your supervisor in the program? | 1. YES  2. NO | | | | | | | | | | 🡪 Q210  🡪 Q211  🡪Q211 |
|  | How did you report the stock out? | 1. In person 2. Through a phone call 3. Through SMS / Text message 4. Using a paper report   999 No answer | | | | | | | | | |  |
| 211 | The last time you experienced a stockout, how many days passed before you were restocked? | __ DAYS PASSED  88. ___ NEVER RESTOCKED  -99 Does not remember / no Response | | | | | | | | | |  |
| 212 | Have you ever borrowed contraceptives from your fellow CBD? | 1. Yes  2. Non | | | | | | | | | |  |
| **Now, I would like to ask you questions about your experience during the insertion of Sayana Press** | | | | | | | | | | | | |
| 213. | Were you anxious / nervous before inserting Implanon NXT the first time?  *Would you say you were…* | 1. Extremely nervous  2. Somewhat nervous  3. A little nervous  4. Not nervous at all  999. Don’t know / Don’t remember.  . | | | | | | | | | |  |
| 214. | What were you worried about when you had to do the injection?  (SELECT ALL THAT APPLY) | 1. Fail to disinfect /prepare the insertion properly  2. Hurt the woman  3. Take too much time to do the insertion  4. Not knowing how to advise the woman on side effects  5. Not appearing confident or professional enough  6. Other: Specify__________  999. No answer | | | | | | | | | |  |
| 215. | Were you more comfortable with the insertions after some practice or were you not more comfortable? | 1. Yes, much more comfortable  2. Yes, somewhat more comfortable  3. No, not much more comfortable  4. No, still not comfortable at all  999. Don’t know / Don’t remember | | | | | | | | | | 🡪 Q216  🡪 Q216  🡪 Q301  🡪 Q301  🡪 Q301 |
| 216 | After how many insertions did you start feeling more comfortable?  (ASK THEM TO ESTIMATE) | _______Number of insertions  999 Does not know / Does not remember | | | | | | | | | |  |
| 217 | During your experience as an Implanon NXT provider, did you encounter the following issues:  Several responses possible.  READ ALL THE ANSWERS AND CHECK ALL THAT APPLY | 1. Woman complaining about pain during the insertion  2. Woman complaining about pain / side effects after the insertion  3. Woman presenting bruises / bleeding at the insertion location  4. Impossible to insert Implanon NXT for a woman  5. Husband / Partner or family have complained  6. Issues if the HZ staff  7. Issues with other health providers (Specify)  8. Verbal / Physical battery while working in the community  9. Other (Specify)  0. No particular problems | | | | | | | | | |  |

**SECTION 3: RELATIONSHIPS WITH OTHERS**

| **Now I would like to ask you about your experience with other persons in relation to your work on the project.** | | | | |  |
| --- | --- | --- | --- | --- | --- |
| **No.** | **QUESTIONS** | | **CATEGORIES** | **SKIP** |  |
| 301 | If a woman in the community prefered a method that you do not provide (for example, Depo or an IUD) instead, what do you tell her?  (DO NOT READ THE ANSWERS BUT CHECK THE ONE GIVEN) | | 1. REFER TO A HEALTH FACILITY (HOSPITAL, CLINIC, HEALTH CENTER) 2. THERE IS NOTHING I CAN DO. 3. I would insist to give her one of the methods I have in stock 4. I would insist to give her Implanon NXT 5. Other (Specify)   999 No response / does not know |  |  |
| 302 | Suppose a woman to whom you have inserted Implanon NXT complains of side effects. What do you tell her?  (DO NOT READ THE ANSWERS BUT CHECK THE ONE GIVEN) | | 1. I tell her to just wait and the effects will go away 2. I refer her to the health facility in the health zone that offers family planning services, where she can consult the nurse regarding her side effects. 3. I refer her to a health facility (no mention of FP services offered) 4. I tell her I will check with my supervisor and get back to her when I know more 5. Other   99 No response / does not know |  |  |
| 303 | Do you know a health facility where nurses have also been trained in inserting Implanon NXT? | | 1. Yes 2. No | 🡪 Q304  🡪 Q308 |  |
| 304 | What is the name of this facility?  (Write the name of the facility) |  | |  | |
| 305 | Do you know the name of the person who has been trained in inserting Implanon NXT in this facility? | 1. Yes   2. No | | 🡪 Q306  🡪 Q307 | |
| 306 | Do you feel that this health facility approves of medical/nursing students providing contraceptives in the community, or they do not approve of this idea? | 1. Completely approves 2. Somewhat approves 3. Somewhat disapproves 4. Completely disapproves   999. Does not know / No response | |  | |
|  | Have you ever referred an Implanon NXT client to a health facility? (Whether or not it offered Implanon NXT) | 1. Yes  2. No | |  | |
| 308 | Why did you refer client(s) to a health facility?  SEVERAL RESPONSES POSSIBLE, CHECK ALL THAT APPLY | 1. Side effects from Implanon NXT  2. Side effects from other methods  3. Client wanted a different method  4. Client preferred to visit the health center / a doctor  5. Other (Specify)  999 Does not know / No response | |  | |
| 309 | In general, what has been the reaction of members of the community to your providing contraceptive services: favorable, unfavorable, or neutral? | 1. Very favorable 2. Somewhat favorable (despite minor resistances) 3. Not very favorable (lots of resistance) 4. Not favorable at all   999 No Response | |  | |
| 310 | Since you began as a community-distributor of contraceptives, has anyone or any group expressed open opposition to your work? | 1. YES 2. NO | | 🡪 Q311  🡪 Q401 | |
| 311 | What was the reason for their opposition? | 1. AGAINST FAMILY PLANNING IN GENERAL 2. Against modern (“artificial”) contraceptive methods 3. Specifically against implants 4. Against having students perform insertions 5. OTHER (Specify:______)   999 No response | |  | |

**SECTION 4: EXPERIENCE AS A COMMUNITY-BASED DISTRIBUTOR**

| **Now, I’d like to ask just a few short questions about your experience providing Implanon NXT today.** | | | |
| --- | --- | --- | --- |
| **NO.** | **QUESTIONS** | **CATEGORIES** | **SKIP** |
| 401 | Overall, what is your level of satisfaction with your experience of being a community-distributor of contraceptives | 1. Very satisfied 2. Somewhat satisfied 3. Somewhat unsatisfied 4. Not satisfied at all   999 Does not know / No opinion |  |
| 402 | What have been the positive aspects (if any) of being a distributor.  DO NOT READ THE QUESTION BUT CHECK ALL THAT APPLY. | 1. GAIN Technical EXPERIENCE RELATED TO MY CAREER (performing injections and insertions, explaining secondary effects) 2. Gain human experience for my carrier (interacting with the community, lead advocacy activities) 3. CONTRIBUTE TO THE WELFARE OF THE COMMUNITY 4. OTHER (Specify)   999 Does not know / No response |  |
| 403 | What have been the negative aspects (if any) of being a distributor?  DO NOT READ THE QUESTION BUT CHECK ALL THAT APPLY. | 1. This activity is time consuming 2. NO/INSUFFICIENT MONETARY COMPENSATION 3. Lack of technical support (injections supplies, reporting forms) 4. Not enough training 5. Not enough monitoring / support from supervisors during the activity 6. Rumors and resistances in the community were frustrating 7. Other (Specify)   - 99 Does not know / No Response |  |
| 404 | Would you recommend another student in medical/nursing school to become involved in this program, or on the contrary, would you discourage him/her to do it? | 1. I would strongly encourage them 2. I would encourage them 3. I would not encourage them 4. I would strongly discourage them |  |
| 405 | Why? |  |  |
| \| **That is all the questions I have for you today. Thank you for taking the time to share your views on Implanon NXT and your role as a distributor. It will help us to understand how to improve the program as it expands.** \| \| --- \| | | | |
