# Supplementary material for: Feasibility and acceptability of using medical and nursing students to provide Implanon NXT at the community level in Kinshasa, Democratic Republic of Congo
Source: BMC Womens Health. 2020 Jun 24;20:133. doi: 10.1186/s12905-020-00993-9 (PMC7315479; doi:10.1186/s12905-020-00993-9)
Supplement: Supplementary file 2 — Additional file 2. [file 12905_2020_993_MOESM2_ESM.docx]

**Appendix B. a. i :** **Initial Acceptor Survey: Implanon NXT**

| Women ID # | \|___\|___\|___\|___\|___\| | Community # | \|___\| | Sensibilization : O/N |
| --- | --- | --- | --- | --- |
| Surveyor ID # | \|___\|___\|___\| | Date: DD/MM/YY | \|___\|___\|___\| | Group : \|___\| |

**SECTION 1: CARACTERISTIQUES DES ACCEPTANTES DE IMPLANON NXT**

| **NO.** | **QUESTIONS ET FILTRES** | **CATEGORIES CODEES** | **SKIP** |
| --- | --- | --- | --- |
|  | How old were you on your last birthday? | AGE IN YEARS: ____________ |  |
|  | Have you ever attended school? | 1. Yes 2. No | 🡪**Q103**  🡪**Q105** |
|  | What is the highest level of education you have completed ? | 1. No schooling 2. Primary 3. Secondary 4. University 5. 99. Other |  |
| 104. | Are you currently attending school? | 1. Yes 2. No | 🡪**Q105**  🡪**Q106** |
| 105. | At what level are you currently studying? | 1. Secondary 2. University 3. Other (Specify) |  |
| 106. | As you know some woman take on a job for which they are paid either in cash or in kind. Others sell things or work in their family farm or tin the family business.  Over the last two years have you ever done one of these things or another type of work? | 1. Yes 2. No | 🡪**Q107**  🡪**Q110** |
| 107 | What is your profession (i.e. what is your main activity / work)? | Profession :________________________ |  |
| 108 | Are you working for someone in your family, for someone else, or are you self-employed? | 1. For a family member 2. For someone else 3. Self-employed |  |
| 109 | Are you paid in cash, kind or are you not paid at all? | 1. In cash only 2. In cash and kind 3. In kind only 4. Not paid |  |
| 110 | Are you currently married or do you live with a man as if you were married? | 1. Yes, civil marriage 2. Yes, customary marriage 3. Yes, religious marriage 4. Yes, lives with a man 5. No, not in a union | 🡪**Q111**  🡪**Q111**  🡪**Q111**  🡪**Q111**  🡪**Q113** |
| 111 | Does your husband / partner currently live with you or does he live elsewhere? | 1. Lives with me 2. Lives elsewhere |  |
| 112 | Does your husband / partner currently holds a permanent job or is he a student? | 1. Yes, has a permanent job  2. Yes, is a student  3. No, none of these |  |
| 113 | Do you have living children? | 1. Yes   No | 🡪 Q114  🡪 Q115 |
| 114 | How many boys and girls? | Number of boys _____________________  Number of girls _____________________ |  |
| 115 | What is your religion?  **DO NOT READ THE ANSWERS**  **If “other” specify on the next screen** | 1. Catholic 2. Protestant 3. Kimbanguist 4. Muslim 5. Pentecostal 6. Evangelical 7. Other (specify):___________________________ |  |
| 116. | What is your province of origin? | \| 1. Bandundu \| \| --- \| \| 1. Bas Congo 2. Equateur 3. Kasai Occidental 4. Kasai Oriental 5. Katanga 6. Kinshasa 7. Maniema 8. Orientale 9. Nord Kivu 10. Sud Kivu \| |  |
| 117. | In your household, do you have   1. Electricity? 2. A radio? 3. A TV? 4. A cell phone? 5. A fridge? 6. A table? 7. A chaise? 8. A couch? 9. A bed? 10. A closet ?   m. A clock? | YES NO DK   1. electricity 1 2 8 2. radio 1 2 8 3. TV 1 2 8 4. cell phone 1 2 8 5. fridge 1 2 8 6. table 1 2 8 7. chair 1 2 8 8. couch 1 2 8 9. bed 1 2 8 10. closet 1 2 8 11. clock 1 2 8 | 1 |
| 118 | Do you own a cell phone for your personal use? | 1. Yes 2. No | 🡪 Q119  🡪 Q201 |
| 119 | Can you receive text messages on your phone? | 1. Yes 2. No   999 Does not know |  |

**SECTION 2: WOMEN AUTONOMY AND DECISION MAKING**

| **No.** | **QUESTIONS AND FILTERS** | **CODING CATEGORIES** | **SKIP** |
| --- | --- | --- | --- |
| **I would like to ask you questions about how some people perceive family planning and how decisions about family planning and other topic are made in your household.** | | | |
| 201. | Would you say that IN YOUR COMMUNITY almost all people, most people, some people or almost no one is in favor of family planning methods? | 1. Almost everyone 2. Most people 3. Some people 4. No one or almost no one   999 Does not know |  |
| 202. | What are the strongest resistances in your community?  Several responses possible  DO NOT READ THE ANSWERS BUT CHECK ALL THAT APPLY | 1. Against limiting number of children (people prefer to have large families) 2. Against using family planning methods in general 3. Against using modern contraceptive methods (condoms, pills, injectables, implants, etc…) 4. Other (Specify):_______   999 Does not know |  |
| 203 | Would you say that AMONG PEOPLE YOU KNOW WELL almost all people, most people, some people or almost no one is in favor of family planning methods? | 1. Almost everyone 2. Most people 3. Some people 4. No one or almost no one   999 Does not know |  |
| 204 | What are the strongest resistances among people you know well?  Several responses possible  DO NOT READ THE ANSWERS BUT CHECK ALL THAT APPLY | 1. Against limiting number of children (people prefer to have large families) 2. Against using family planning methods in general 3. Against using modern contraceptive methods (condoms, pills, injectables, implants, etc…) 4. Other (Specify):_______ 5. 999 Does not know |  |
| **205.** | When you went to receive family planning services today, were you concerned that someone might see you and guess Would you say you were very concerned, somewhat concerned or not concerned at all? | 1. Yes, very concerned  2. Yes, somewhat concerned  3. No, not concerned at all  999 No response |  |
| **206** | (Check Q110)  Does your husband / partner know that you came to the family planning consultation today? | 1. Yes  2. No | 🡪 Q207a  🡪 Q207b |
| **207.** | 207a. Does your husband / partner agree for you to use a family planning methods?  207b. Do you think your husband / partner would agree for you to use a family planning method? | 1. Yes, he is / would completely agree 2. Yes, he is / would rather agree 3. No, he is / would rather disagree 4. No, he is not / completely disagree   99 9 Does not know |  |
| **208.** | How did you hear about the contraceptive distribution activity that is taking place today?? | 1. Through the health center staff during a consultation 2. Through outreach activities in the community 3. Through friends / neighbors / family 4. Through my husband / partner 5. Through public signs / radio emissions 6. Other (Specify) :___________   999 Does not know / Does not remember |  |
| **209.** | Who influenced your decision to come to the family planning consultation today?  Several responses possible  DO NOT READ THE ANSWERS BUT CHECK ALL THAT APPLY | 1. Only me 2. My husband / partner 3. My husband / partner and I (joint decision) 4. Healthcare providers 5. Community Based Distributors 6. Friends / Neighbors / Family 7. Other (Specify):_______   999 Does not know |  |

**SECTION 3: CONTRACEPTIVE HISTORY, PREFERENCES AND USE OF FP SERVICES**

| **Now I would like to ask you questions about your past experience using FP services** | | | | |
| --- | --- | --- | --- | --- |
| **No.** | **QUESTIONS** | | **CATEGORIES** | **SKIP** |
| 301 | Before today, have you ever used a method or done something to delay or avoid becoming pregnant? | | 1. Yes 2. No | 🡪**Q303**  🡪**Q306** |
| 303 | Which methods have you used in the past to avoid becoming pregnant? Have you or your partner ever used?  READ ALL THE OPTION AND CHECK Yes or NO   1. Male Sterilization? 2. The daily pill? 3. The IUD/coil? 4. Implant? 5. Male Condoms? 6. Female Condoms? 7. Emergency Contraception/EC/Emergency pill? 8. Lactational Amen. Method? 9. CycleBeads? 10. Withdrawal? 11. Injectable? 12. Something else? | | **ENREGISTREZ TOUTES LES REPONSES.**   1. Male Sterilization? Y/N 2. The daily pill? Y/N 3. The IUD/coil? Y/N 4. Implant? Y/N 5. Male Condoms? Y/N 6. Female Condoms? Y/N 7. Emergency Contraception/EC/Emergency pill? Y/N 8. Lactational Amen. Method? Y/N 9. CycleBeads? Y/N 10. Withdrawal? Y/N 11. Injectable? Y/N   Something else? Y/N |  |
| 304 | What is the *most recent* contraceptive method that you have used before today?? | | 1. Male condom 2. Daily pill 3. Injectables 4. Implant 5. IUD 6. Male sterilization 7. Female condom 8. Emergency contraception 9. MAMA 10. Cyclebeads 11. Withdrawal? 12. Something else: ________ |  |
| 305 | Why did you decide on that method?  **DO NOT READ RESPONSES. RECORD ALL SPONTANEOUSLY MENTIONED**.  IF “OTHER”, SPECIFY ON NEXT SCREEN | 1. PREVENTS PREGNANCY EFFECTIVELY 2. MORE EFFECTIVE THAN PREVIOUS METHOD 3. METHOD IS DOES NOT PRESENT HEALTH RISK 4. PROVIDER RECOMMENDED IT 5. HUSBAND/PARTNER RECOMMENDED IT 6. FRIENDS, RELATIVES RECOMMENDED IT 7. AVAILABLE/EASY TO OBTAIN 8. CONVENIENT TO USE 9. FEW SIDE EFFECTS 10. CAN EASILY HIDE IT 11. AFFORDABILITY 12. OTHER (Specify): _________________________ | |  |
| 306 | Are you aware that some community health workers (CHW) offer certain family planning methods in the community? | 1. YES 2. NO | | 🡪Q307  🡪Q309 |
| 307 | Have you ever visited a CHW to obtain family planning methods? | 1. YES 2. NO | | 🡪**Q308**  🡪**Q309** |
| 308 | Which family planning methods did you receive from a CHW?  IF “OTHER”, SPECIFY ON NEXT SCREEN | 1. PILLS  2. CONDOMS  3. CYCLEBEADS  4. OTHER____________ | |  |
| 309 | If you had the choice in which location would you prefer to receive family planning services? | 1. Visit at the clinic / the health center 2. With a CBD in a community place (center, market, church) 3. With a CBD coming to your house   99. No prference | | 🡪**Q314a**  🡪**Q314B**  🡪**Q314C**  **🡪Q401** |
| 310 | q310a. Why would you prefer to visit a clinic / health center?  q310b. Why would you prefer to visit a CBD in a community place?  q310c. Why would you prefer to be visited by a CBD at hour house?    **DO NOT READ RESPONSES. RECORD ALL SPONTANEOUSLY MENTIONED.**  **IF “OTHER”, SPECIFY IN NEXT SCREEN** | 1. EASY/CONVENIENT LOCATION 2. COSTS LESS FOR METHOD OR TRAVEL 3. DON’T HAVE TO WAIT/AVOID LINES 4. VISITING FOR OTHER REASONS ANYWAY 5. HAVE CONFIDENCE IN PROVIDER/CHW 6. KNOW/LIKE THE PROVIDER/CHW PERSONALLY 7. MORE DISCRETE, PRIVATE 8. PREFERRED METHOD AVAILABLE 9. FEWER STOCK OUTS 10. OTHER (Specify):_______________________ | |  |

**SECTION 4: INITIAL IMPLANON NXT INSERTION EXPERIENCE**

| **Now, I’d like to ask just a few short questions about your experience receiving Implanon NXT from the provider today.** | | | | |
| --- | --- | --- | --- | --- |
| **NO.** | **QUESTIONS** | **CATEGORIES** | | **SKIP** |
| 401 | Were you anxious before receiving Implanon NXT today?  Would you say that you were very anxious, somewhat anxious, or not anxious at all? | 1. VERY ANXIOUS 2. A LITTLE BIT ANXIOUS 3. NOT AT ALL ANXIOUS   999 No answer | | 🡪**Q402**  🡪**Q402**  **🡪 Q403** |
| 402 | What concerns did you have?  **DO NOT READ RESPONSES. RECORD ALL SPONTANEOUSLY MENTIONED** | 1. Concerns about its effectiveness to prevent pregnancies 2. Fear of side effects / health issues 3. Fear of future sterility 4. Fear of birth defects 5. Pain during or after the insertion 6. More familiar with other methods 7. Husband / partner opposition 8. Other (specify):_________ | |  |
| 403 | Today, did you attend a group talk on FP method, received individual counseling, both or neither of these? | 1. Group talk on FP methods only 2. Individual counseling only 3. Both 4. Neither of these   999 No answer | |  |
| 404 | How much time did pass between the moment you arrived here and the moment the CBD came to talk to you (approximately)? | _____ Hours _________ Minutes | |  |
| 405 | How much time did you consultation with the CBD last (approximately)?  (Between the moment he / she came to meet you and the moment you left him / her) | _____ Hours _________ Minutes | |  |
| 406 | Overall, would you say that your visit (wait time + consultation) took too much time, an adequate amount of time, or not enough time? | 1. Too much time  2. An adequate amount of time  3. Not enough time  999. Does not know / No answer | |  |
| 407 | Did you get the impression that the CBD was comfortable in preparing and completing the insertion?  Would you say they seemed completely comfortable, somewhat comfortable, not very comfortable or not comfortable at all? | 1. Yes, completely comfortable  2. Yes, somewhat comfortable  3. No, not very comfortable  4. No, not at all comfortable  999. Does not know / No answer | |  |
| 408 | What level of pain did you feel *during* the insertion? Was it painless or nearly painless, a little painful or very painful? | 1. PAINLESS OR NEARLY PAINLESS 2. A LITTLE PAINFUL 3. VERY PAINFUL   999. Does not know / No answer | |  |
| 409 | What level of pain did you feel *a few minutes after* the insertion? Was it painless or nearly painless, a little painful or very painful? | 1. PAINLESS OR NEARLY PAINLESS 2. A LITTLE PAINFUL   VERY PAINFUL | |  |
| 410 | Do you notice any immediate skin irritation from the initial insertion of Implanon NXT? | 1. YES 2. NO | | 🡪**Q411**  🡪**Q412** |
| 411 | How would you rate/classify this skin irritation? | 1. MILD 2. MODERATE 3. SEVERE | |  |
| 412 | Before receiving Implanon NXT today, were you aware that the CBD providing it was a medical / nursing school student? | 1. Yes  2. No | | **🡪 Q413**  **🡪 Q414** |
| 413 | How comfortable were you with having a medical / nursing school student complete the insertion rather than a doctor or a nurse? | 1. Very comfortable  2. Somewhat comfortable  3. Not very comfortable  4. Not comfortable at all  999 No answer | |  |
| 414 | In this appointment, how well did your CHW provider explain to you possible side effects of Implanon NXT and where to get help if needed? | 1. EXPLAINED WELL 2. EXPLAINED ADEQUATELY 3. EXPLAINED POORLY 4. DID NOT EXPLAIN AT ALL 5. DON’T KNOW | |  |
| 413 | Did the CBD tell you where to go / who to contact if you have questions or if you’re experiencing side effects? | 1. Yes  2. No  999 Does not know / Cannot remember | |  |
| 414 | Currently, who would you contact / where would you go if you had questions regarding Implanon NXT or if you were experiencing side effects?  Several answers possible  **DO NOT READ RESPONSES. RECORD ALL SPONTANEOUSLY MENTIONED.** | 1. CBD 2. The healthcare center recommended by the CBD 3. Other healthcare provider 4. Family, friends, neighbors 5. Husband / Partner 6. Other: ________________   999 Does not know | |  |
| 415 | All things considered, what is your level of satisfaction with the *information and counseling* you’ve received from the student who provided you the Implanon NXT and other contraceptive options? | 1. VERY SATISFIED  2. SOMEWHAT SATISFIED  3. SOMEWHAT UNSATISFIED  4. VERY UNSATISFIED  999. Does not know / No answer | |  |
| 416 | How satisfied are you with *insertion process* completed by the student? | 1. VERY SATISFIED  2. SOMEWHAT SATISFIED  3. SOMEWHAT UNSATISFIED  4. VERY UNSATISFIED  999. Does not know / No answer | |  |
| 417 | All things considered, what is your *global level of satisfaction* with the service you have received from the student who provided you the Implanon NXT? | 1. VERY SATISFIED  2. SOMEWHAT SATISFIED  3. SOMEWHAT UNSATISFIED  4. VERY UNSATISFIED  999. Does not know / No answer | |  |
| 418 | Did you receive Implanon NXT for free (during a campaign day) or did you pay something to obtain this method? | 1. Received for free  2. Paid something  99 Does not remember | |  |
| 419 | How much did you pay to receive Implanon NXT? | Amount in Congolese Francs: _________ XOF | |  |
| 420 | What would be, according to you, a reasonable price that women in your community would be ready to pay to receive Implanon NXT? | Amount in Congolese Francs: _________ XOF | |  |
| 421 | Currently, would you recommend Implanon NXT to a friend who would like to avoid becoming pregnant? | 1. I would highly recommend it 2. I would recommend it 3. I would not recommend it   99 Indifferent / Does not know | |  |
| **We are at the end of the questionnaire. Thank you for taking the time to answer my questions today. We would like to re-contact you in six month to plan a second interview. We would like to ask you if you’d agree to be contacted to set up an appointment for this interview. Once again, you are under no obligation to participate in this study and you will be able to receive services from a CBD even if you do not wish to answer our questions. We will only contact you in six month if you give us your permission today. If you do not want to give us permission to contact you later, then your participation in this study will end now.** | | | | |
| 422 | Would you accept to be contacted for a third interview in six month? | | 1. Yes 2. No | 🡪Q423  🡪 End |
| **423** | Would you prefer this interview to take place at your house, in this very same place, or in a different place? | | 1. At home 2. Same place 3. Different place (specify): | 🡪Q424  🡪Q425  🡪Q424 |
| **424** | What is your address? | | Address: |  |
| **425** | What is the best phone number to contact you in order to schedule the interview? | | Phone number______________________ |  |
| **426** | If we cannot reach you by phone, is there another person we can call? We will not tell her / him why, but simply that the health center wants to re-contact you? | | Name of secondary contact:  Phone number of secondary contact: |  |
| **427** | This ID card will help us connect the information you gave us today with your answers during the follow-up interview. This will help us to better understand your situation at the end of the pilot study.  Thanks for bringing it with you when you will come by in 6 month.  ***The surveyor must write down the date of the follow-up survey on the ID Card before giving it to the woman.***  ***Write down the ID Card Number below*** | | **NXT_R1**  DD/MM/YY \|___\|___\|___\|___\|___\|  Woman ID Number \|___\|___\|___\| |  |
| **This is all for today. Thank you for helping us with this research. I will contact you at the address / number you provided us in about three month to ask you other questions about your experience. Until then, if you have questions or concerns, do not hesitate to contact me, or to contact the clinic / healthcare provider.** | | | | |
