# Supplementary material for: Feasibility and acceptability of using medical and nursing students to provide Implanon NXT at the community level in Kinshasa, Democratic Republic of Congo
Source: BMC Womens Health. 2020 Jun 24;20:133. doi: 10.1186/s12905-020-00993-9 (PMC7315479; doi:10.1186/s12905-020-00993-9)
Supplement: Supplementary file 3 — Additional file 3. [file 12905_2020_993_MOESM3_ESM.docx]

**Appendix B.b.i : Follow-up interview with customers 6 month after the insertion**

| ID Card # | \|___\|___\|___\|___\|___\| |
| --- | --- |
| Initial interview date : JJ/MM/AA | \|___\|___\| / \| ___\|___\| / \|___\|___\| |

| Women ID # | \|___\|___\|___\|___\|___\| | Community # | \|___\| | Sensibilization : O/N |
| --- | --- | --- | --- | --- |
| Surveyor ID # | \|___\|___\|___\| | Date: DD/MM/YY | \|___\|___\|___\| | Group : \|___\| |

| NO. | **QUESTIONS** | **CODE** | | **SKIP** |
| --- | --- | --- | --- | --- |
| **To begin, I’d like to ask you a few questions about you and your family. I know you have already answered these questions before but we need these information to better understand your situation today.** | | | | |
| **101** | How old were you on your last birthday? | AGE IN YEARS: ____________ |  | |
| **102** | What is the highest level of education you have completed ? | 1. No schooling 2. Attended primary school 3. Completed primary school 4. Attended secondary school 5. Completed secondary school 6. Attended university 7. Completed university   99. Other |  | |
| **103** | Are you currently married or do you live with a man as if you were married? | 1. Yes, civil marriage 2. Yes, customary marriage 3. Yes, religious marriage 4. Yes, lives with a man 5. No, not in a union |  | |
| **104** | Do you have living children? | 1. No 2. Yes   Number of children: ____ |  | |
| **105** | What is your religion?  **DO NOT READ THE ANSWERS**  **If “other” specify on the next screen** | 1. Catholic 2. Protestant 3. Kimbanguist 4. Muslim 5. Pentecostal 6. Evangelical 7. Other (specify) 8. ___________________________ |  | |
|  | | | | |
| **NO.** | **QUESTIONS** | **CODE** | | **ALLER A** |
| **Remember, six month ago, when you received your insertion of Implanon NXT in the community. I would like to ask you a few questions about what happened and your experience during this first appointment to receive the insertion.** | | | | |
| **106** | Have you indeed received an insertion of Implanon NXT six month ago in your community?? | 1. Yes  2. No | 🡪**Q107**  🡪 The woman is not eligible. Go to the end of the questionnaire | |
| **107** | Where / in Which location did you receive this insertion? | 1. At the healthcare center 2. At home 3. In a community place (church, market, etc) 4. At school / on the university campus 5. Other (specify) |  | |
| **108.** | After the first insertion, did you notice a skin irritation / allergic reaction ? Did you notice a problem where the implants was inserted? | 1. Yes  2. No | 🡪**Q108**  🡪**Q114** | |
| **109.** | What type of reaction did you notice??  Several responses possible  **DO NOT READ THE RESPONSES BUT CHECK ALL RESPONSES SPONTANEOUSLY MENTIONED.** | 1. Sensibility / Pain 2. Bruise / Discoloration 3. Swelling 4. Scar 5. Nodule / Bump under your skin 6. Blister 7. Fluid leaking 8. Other (specify on the next screen): _________________________ |  | |
| **110.** | Did you visit a hospital, a clinic, a CBD or a pharmacy, or did you contact anyone to receive counseling or a treatment? | 1. Yes  2. No | 🡪**Q111**  🡪**Q112** | |
| **111.** | Who did you contacts for this counseling or treatment? | 1. Hospital / Clinic 2. CBD 3. Pharmacist / Store 4. Research team 5. Someone else (specify):___ |  | |
| **112.** | Did the reaction disappear, either on its own or after treatment? | 1. Yes, disappeared after treatment 2. Yes, disappeared on its own 3. No   999 Does not know |  | |
|  | **Observe the insertion spot and check if the problem needs to be addressed**  **Refer to a doctor if necessary** | | | |
| **113.** | Did you experience side effects using Implanon NXT | 1. Yes 2. No | 🡪**Q114**  🡪**Q119** | |
| **114.** | Which side effects did you experience?  Several responses possible  **DO NOT READ THE RESPONSES BUT CHECK ALL RESPONSES SPONTANEOUSLY MENTIONED.** | 1. Irregular period 2. No periods 3. Abundant or frequent periods 4. Weight gain 5. Weight loss 6. Back pain 7. Headaches 8. Stomach pain 9. Nausea / Vomitting 10. Lack of libido 11. Vaginal dryness 12. Pain at the location of the insertion 13. Mood swings 14. Weakness or fatigue 15. Breast pain 16. Acne 17. Other (specify):_________________ |  | |
| **115.** | Did you try to receive counseling or treatment for your side effects? | 1. Yes 2. No | 🡪**Q116**  🡪**Q118** | |
| **116.** | Who did you contacts for this counseling or treatment? | 1. Hospital / Clinic 2. CBD 3. Pharmacist / Store 4. Research team 5. Someone else (specify):___ |  | |
| **117.** | Did you receive a treatment? | 1. Yes 2. No |  | |
| **118.** | Did the side effects disappear, either on their own or after treatment? | 1. Yes, disappeared after treatment 2. Yes, disappeared on their own 3. No   999 Does not know |  | |
| **119.** | Since you have received Implanon NXT, did you get pregnant? | 1. Yes  2. No  999 Does not know |  | |
| **120** | Does your husband / partner know that you are using Implanon NXT? | 1. Yes 2. No   99. Not sure / Does not know |  | |
| **121** | Does your husband / partner agree with your using Implanon NXT to prevent pregnancies?  Would you say he completely agrees, somewhat agrees; somewhat disagrees, or completely disagrees? | 1. Completely agrees 2. Somewhat agrees 3. Somewhat disagrees 4. Completely disagrees |  | |
| **Now that we have talked about your first insertion, I would like to ask you about your impressions regarding the product in general and your intentions to use it or not in the future?** | | | | |
| **201** | Did you have the Implanon NXT removed or is it still in place? | - - - 1. Removed   2. Still in place | | **🡪Q202**  **🡪Q203** |
| **202** | 202a. Why did you choose to have the implant removed? | 1. Side effect  2. Husband / Partner was opposed  3. Preference for another method  4. Wanted to get pregnant  5. Other (Specify): _______  999. No response | |  |
|  | 202b. Where did you have the implant removed | 1. Health center 2. Hospital 3. CBD 4. Other (Specify):________ | |  |
|  | 202c. Did you have any issue when the implant was removed? | 1. Yes (Specify):______ 2. No | |  |
|  | 202d. À Do you plan to use Implanon NXT again in the future?? | 1. Yes 2. No | | 🡪**Q204**  **🡪Q205** |
| **203** | Currently, do you plan to continue using Implanon NXT, to use a different contraceptive method, or to not use any contraceptive method for the moment? | - - - 1. I plan on continuing to use Implanon NXT       2. I plan on using a different method       3. I plan on not using any contraceptive method for the moment       4. Does not know / Is not sure | | **🡪Q204**  **🡪Q205**  **🡪Q205**  **🡪Q206** |

| **204** | Why would you chose to keep using Implanon NXT rather than another method?  **DO NOT READ THE RESPONSES BUT CHECK ALL RESPONSES SPONTANEOUSLY MENTIONED.** | 1. Easy to use 2. It’s very effective 3. Wants to be protected longer 4. Less painful than other methods 5. Few side effects 6. Easy to hide 7. No need to go to the health center 8. No need to remember the dates 9. Other (Specify on the next screen)   99. No response |  |
| --- | --- | --- | --- |
| **205** | Why did you not chose / would you not chose to use Implanon NXT?  **DO NOT READ THE RESPONSES BUT CHECK ALL RESPONSES SPONTANEOUSLY MENTIONED.** | 1. Concerns about its effectiveness to prevent pregnancies 2. Fear of side effects / health issues 3. Fear of future sterility 4. Fear of birth defects 5. Pain during or after the insertion 6. More familiar with other methods 7. Husband / partner opposition 8. Wants to become pregnant 9. Other (Specify):______ |  |
| **206** | Currently, would you recommend Implanon NXT to a friend who would like to avoid becoming pregnant? | 1. I would highly recommend it 2. I would recommend it 3. I would not recommend it   99 Indifferent / Does not know |  |
| **207** | Do you know how long Implanon NXT protects you from pregnancies? | _______ Number   1. Weeks 2. Months 3. Years |  |
| **Now, I would like to talk about your experience with the CBD who inserted Implanon NXT six months ago** | | | |
| **301** | When you received Implanon NXT six months ago, did you know that the person who inserted it was a medical / nursing school student? | 1. Yes 2. No |  |
| **302** | How did you know that the person was a medical / nursing school student? | 1. The CBD told me him- / herself when they introduced themselves 2. The supervisor told me when I arrived at the site 3. The Community Worker who did the outreach told me 4. Other (Specify on the next screen)   99. Does not remember / No response |  |
| **303** | How comfortable were you with having a student (as opposed to a doctor or a nurse) performing the insertion? | 1. Completely comfortable 2. Somewhat comfortable 3. Somewhat uncomfortable 4. Completely uncomfortable   99. No response |  |
| **304** | Would you say the student spent not enough time, enough time, or too much time with you during your first appointment? | 1. Too much time 2. Just enough time 3. Not enough time   99. No response |  |
| **305** | Were the explanations given by the student / CBD clear / easy to understand, or were they not clear / hard to understand? | 1. Completely clear 2. Somewhat clear 3. Somewhat unclear 4. Completely unclear   99. No response |  |
| **306** | Did the student / CBD appear comfortable with making the insertion or was he / she uncomfortable? | 1. Completely comfortable 2. Somewhat comfortable 3. Somewhat uncomfortable 4. Completely uncomfortable   99. No response |  |
| **307** | Was the CBD open-minded and respectful towards you or was he / she not open-minded and disrespectful? | 1. Yes, completely open-minded and respectful 2. Yes, somewhat open-minded and respectful 3. No, somewhat not open-minded and not respectful 4. No, completely not open-minded and not respectful   99. No response |  |
| **308** | As you may know, CBD are currently authorized to provide Implanon NXT in the community.  Right now, would you recommend to a friend to receive Implanon NXT from a medical / nursing school student? | 1. Yes, I would recommend it without hesitation 2. Yes, I would rather recommend it 3. No, I would rather not recommend it 4. No, I would absolutely not recommend it   99. No response |  |
|  |  |  |  |
| **We are at the end of the questionnaire. Thank you for taking the time to answer my questions today and thank you for taking the time to participate in this study. We hope that the results will demonstrate that Implanon NXT is a good option for women in DRC. If that is the case, more women could have access to this method in the future.** | | | |
